# Isolated Fe Single Atomic Sites Anchored on Highly Steady Hollow Graphene Nanospheres as an Efficient Electrocatalyst for the Oxygen Reduction Reaction

**DOI:** 10.1002/advs.201801103

**Published:** 2018-11-26

**Authors:** Xiaoyu Qiu, Xiaohong Yan, Huan Pang, Jingchun Wang, Dongmei Sun, Shaohua Wei, Lin Xu, Yawen Tang

**Affiliations:** ^1^ Jiangsu Key Laboratory of New Power Batteries Jiangsu Collaborative Innovation Center of Biomedical Functional Materials School of Chemistry and Materials Science Nanjing Normal University Nanjing 210023 China; ^2^ School of Chemistry and Chemical Engineering Yangzhou University Yangzhou 225009 China

**Keywords:** 3D graphene, nonprecious metals, oxygen reduction reaction, single Fe atoms

## Abstract

The rational design of economical and high‐performance nanocatalysts to substitute Pt for the oxygen reduction reaction (ORR) is extremely desirable for the advancement of sustainable energy‐conversion devices. Isolated single atom (ISA) catalysts have sparked tremendous interests in electrocatalysis due to their maximized atom utilization efficiency. Nevertheless, the fabrication of ISA catalysts remains a grand challenge. Here, a template‐assisted approach is demonstrated to synthesize isolated Fe single atomic sites anchoring on graphene hollow nanospheres (denoted as Fe ISAs/GHSs) by using Fe phthalocyanine (FePc) as Fe precursor. The rigid planar macrocycle structure of FePc molecules and the steric‐hindrance effect of graphene nanospheres are responsible for the dispersion of Fe–N*_x_* species at an atomic level. The combination of atomically dispersed Fe active sites and highly steady hollow substrate affords the Fe ISAs/GHSs outstanding ORR performance with enhanced activity, long‐term stability, and better tolerance to methanol, SO_2_, and NO*_x_* in alkaline medium, outperforming the state‐of‐the‐art commercial Pt/C catalyst. This work highlights the great promises of cost‐effective Fe‐based ISA catalysts in electrocatalysis and provides a versatile strategy for the synthesis of other single metal atom catalysts with superior performance for diverse applications.

## 1 Introduction

The electrocatalytic oxygen reduction reaction (ORR) has been recognized as the key electrochemical process in a range of sustainable energy‐conversion devices, such as fuel cells and metal–air batteries.^1^, ^2^ The sluggish 4‐electron‐involved kinetics and high overpotential of cathodic ORR greatly restrict the energy‐conversion efficiency and remain primary technical challenges hampering the widespread commercialization of these ORR‐involved devices.^3^, ^4^ As is well known, the current commercial high‐performance ORR electrocatalysts still predominantly rely on precious platinum group metals (PGMs), which inherently suffer from prohibitive cost, poor long‐term stability, methanol crossover effect, and vulnerability to poisonous species.^5^, ^6^ In this context, the development of cost‐effective, ultrastable, and high‐efficiency earth‐abundant metal catalysts which can substitute PGM is extremely urgent and thereby has stimulated tremendous research interests in renewable energy field.^7^, ^8^, ^9^, ^10^ Fortunately, a mass of recent studies have demonstrated that Fe–N*_x_*‐based carbon nanomaterials represent one of the most promising alternatives to PGM‐based catalysts for ORR because of their extraordinary catalytic performance, together with a relatively cheap price and abundant reserves.^11^, ^12^

In spite of the controversial identifications of active sites for Fe–N*_x_*‐based carbon catalysts in scientific community, there comes a consensus in catalysis that the reactivity would be remarkably improved by downsizing the catalyst to increase the number of active sites.^13^, ^14^, ^15^, ^16^ Due to the low coordination, maximum atom utilization efficiency, and most exposed active sites, isolated single atom (ISA) catalysts usually exhibit exceptional catalytic performances and thereby have triggered considerable interests.^17^, ^18^, ^19^, ^20^ Nevertheless, the fabrication of ISA catalysts remains a grand challenge due to the very high surface energy, easy migration, and serious aggregation of ISAs during catalytic reaction.^21^, ^22^ One of the effective solutions to ameliorate this issue is anchoring the ISAs on an appropriate substrate, which should be conductive and redox‐inert. Among various substrates, 2D graphene‐based nanosheets are regarded as a class of promising candidate because of their large surface area, outstanding mechanical robustness and superior electrical conductivity.^23^, ^24^, ^25^ Unfortunately, the irreversible stacking of 2D graphene nanosheets would diminish the accessible surface, bury the catalytically active sites and obstruct the mass diffusion/transport, thereby posing a negative effect to electrocatalytic performance.^26^, ^27^, ^28^ To this end, we have recently developed a sophisticated and universal template‐engaged strategy to fabricate 3D graphene hollow nanospheres, which could exhibit improved mechanical strength, accelerated reaction kinetics, and enhanced electrocatalytic performance when served as a catalyst support.^29^, ^30^, ^31^ Therefore, 3D graphene hollow nanospheres are deservedly expected as an ideal platform to immobilize isolated Fe single atoms.

Herein, we for the first time fabricate atomically dispersed Fe–N*_x_* species anchoring on graphene hollow nanospheres (denoted as Fe ISAs/GHSs) by using SiO_2_ nanosphere as sacrificial templates and Fe phthalocyanine (FePc, Figure S1, Supporting Information) as Fe–N*_x_* source. The unique rigid planar macrocycle structure of FePc molecules and π–π interaction between FePc and SiO_2_@GO nanosphere are responsible for the dispersion of Fe–N*_x_* species at an atomic level. The deliberate integration of atomically dispersed Fe active sites with highly steady hollow substrate endows the synthesized Fe ISAs/GHSs an exceptional ORR performance with greatly improved activity, long‐term stability, and better tolerance to methanol, SO_2_, and NO*_x_* in alkaline medium, surpassing commercial 20 wt% Pt/C catalyst and most of the previously reported Fe‐based catalysts. Therefore, the synthesized Fe ISAs/GHSs are a competitive nonprecious ORR electrocatalyst to substitute Pt for renewable fuel cells and metal–air batteries in the future.

## 2 Results and Discussion

### 2.1 Characterization of the Samples

**Figure 1:**
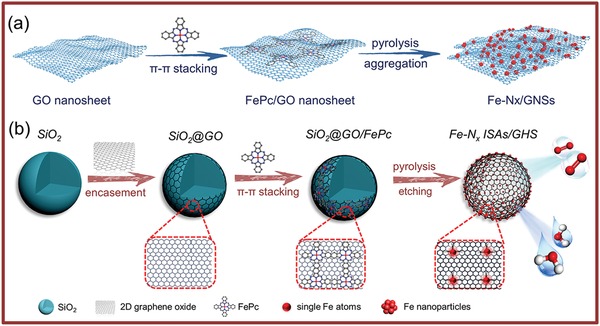
Schematic illustration showing the effects of the dimensionality of graphene substrate on the formation of Fe single atoms. a) FePc molecules supported on 2D graphene nanosheets usually undergo sintering during pyrolysis, and b) FePc molecules supported on 3D graphene hollow nanospheres facilitate the formation of Fe single atoms.

In this work, FePc has been employed as Fe source mainly for the following reasons. First, FePc molecules with rigid macrocycle structures are readily dispersed on the surface of GO through π–π stacking interactions.^32^, ^33^, ^34^ Second, the rigid planar structure of FePc molecules effectively facilitates the isolation of one Fe–N site from another and protect the iron species against diffusion and aggregation upon pyrolysis at high temperature, promoting the dispersing of electrocatalytically active sites. Thirdly, FePc is easily prepared and relatively cheap. It is found that the 3D graphene hollow nanospheres play a decisive role for the formation of Fe single atom catalyst. As schematically shown in **Figure** **1**a, the pyrolysis/deoxygenation of the 2D GO are prone to stack and aggregate during the reaction, resulting in reduced surface area, buried active sites, and impeded mass transfer. Meanwhile, the FePc molecules anchored 2D GO usually induces particle sintering and aggregation during the pyrolysis, imposing a negative effect to catalysis. To overcome the above limitations, 3D graphene hollow nanospheres are employed as a scaffold to immobilize Fe single atoms. Figure 1b schematically illustrates the major synthesis procedure of Fe–N*_x_* ISAs/GHSs using 3D graphene hollow nanospheres as a support. Briefly, presynthesized uniform SiO_2_ nanospheres of 160 nm in diameter (Figure S2a1,a2, Supporting Information) were employed as sacrificial templates to define the formation of graphene hollow nanospheres. Upon surface modification with positive charges, the SiO_2_ nanospheres could be readily encapsulated by GO nanosheets with negatively charged surface and high flexibility via electrostatic attractions, forming SiO_2_@GO core–shell nanospheres (Figure S2b1,b2, Supporting Information). As the introduction of appropriate amount of FePc, FePc molecules could be simultaneously adsorbed on the surface of the SiO_2_@GO nanospheres through π–π stacking interactions, generating SiO_2_@GO/FePc nanospheres (Figure S2c1,c2 and Figure S3, Supporting Information). After being pyrolyzed at 700 °C under N_2_ atmosphere, FePc molecules on SiO_2_@GO/FePc nanospheres would be transformed into N‐doped carbon. Meanwhile, Fe(III) moieties within FePc molecules would be carbothermally reduced by the as‐generated carbon species, producing highly isolated Fe atoms firmly immobilized on nitrogen species (Figure S2d1,d2, Supporting Information). Finally, the inner SiO_2_ nanosphere templates were selectively etched away by NaOH solution, leading to the formation of Fe–N*_x_* ISAs/GHSs.

**Figure 2:**
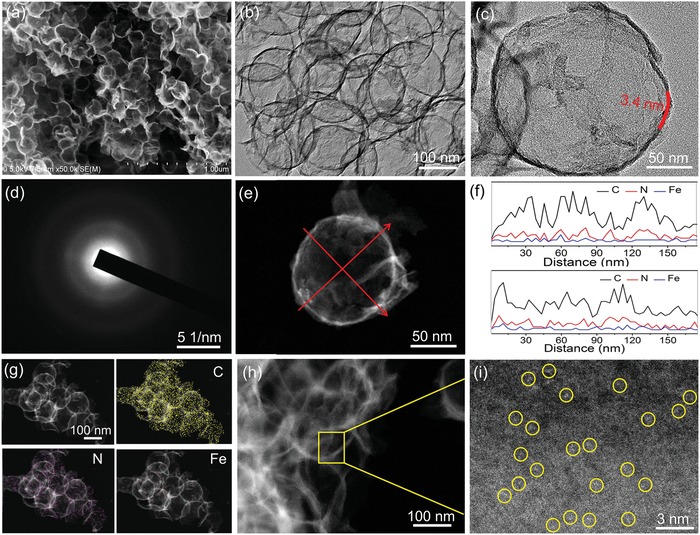
Morphological and compositional analyses of the resultant Fe–N*_x_* ISAs/GHSs. a) SEM image, b) TEM image, c) HRTEM image, d) SAED pattern, e) HAADF‐STEM image, f) EDX line‐scan profiles, g) elemental mapping images, h,i) AC‐HAADF‐STEM images.

The panoramic scanning electron microscopy (SEM) image (**Figure** **2**a) reveals that the resultant Fe–N*_x_* ISAs/GHSs faithfully inherit the initial spherical shape and size of the starting SiO_2_ nanospheres. Additionally, some broken nanospheres with open mouths, hollow cavities, and exposed interior surface can be observed, which could endow a shortened pathway for mass transport and facilitated electrolyte permeability. Impressively, the spherical skeleton of the prepared Fe–N*_x_* ISAs/GHS can still be well preserved without architectural collapse after removing the SiO_2_ templates, suggesting their outstanding mechanical strength. Transmission electron microscopy (TEM) image (Figure 2b) further confirms the complete removal of the SiO_2_ templates and hollow feature of the obtained Fe–N*_x_* ISAs/GHSs. High‐resolution TEM (HRTEM) image (Figure 2c) of an individual Fe–N*_x_* ISAs/GHS indicates that the wall thickness of the hollow nanospheres is approximately 3.4 nm. Moreover, no noticeable metal nanoparticles can be observed, indicating that Fe atoms might exist in an atomically dispersed form. The ring‐like selected area electron diffraction (SAED) pattern (Figure 2d) reveals the poor crystallinity of the as‐prepared Fe–N*_x_* ISAs/GHS. The high‐angle annular dark‐field scanning transmission electron microscope (HAADF‐STEM) image (Figure 2e) also verifies the structural features as hollow nanospheres composed by 3D graphene. The cross‐sectional compositional line profiles (Figure 2f) along different directions and elemental mapping images (Figure 2g) indicate that the trace amount of Fe element is homogeneously distributed over the entire hollow nanospheres. The aberration‐corrected HAADF‐STEM (AC‐HAADF‐STEM) images (Figure 2h,i) further corroborate the formation of isolated Fe single atoms. As marked by yellow circle in Figure 2i, numerous of bright dots with size of around 0.3 nm could be attributed to well‐dispersed Fe atoms because of the different *Z*‐contrast between heavier Fe and lighter C and N. Inductively coupled plasma atomic emission spectroscopy indicates that the weight fraction of Fe in the Fe–N*_x_* ISAs/GHSs is 1.2 wt%.

**Figure 3:**
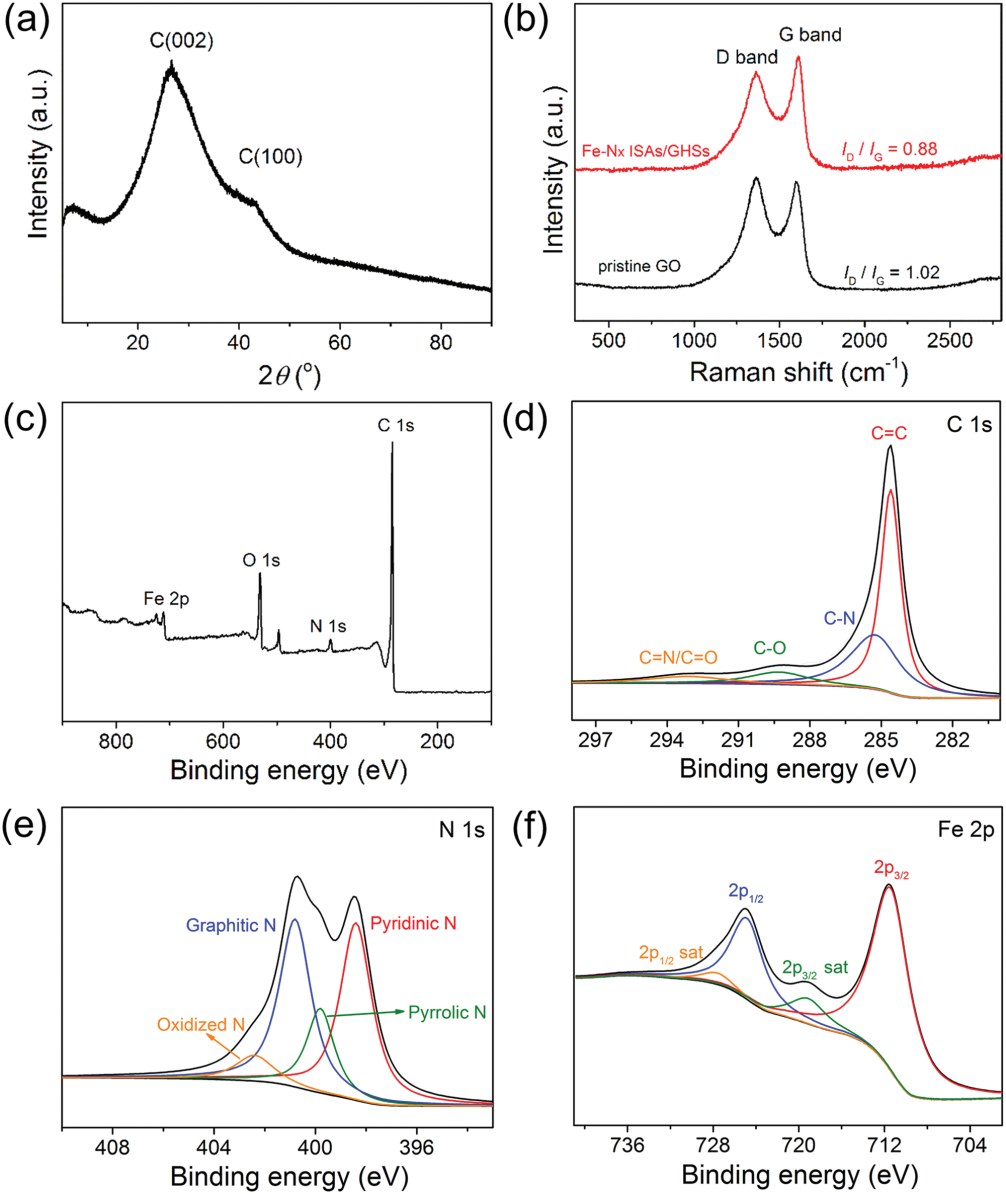
Structural and compositional examinations of the as‐prepared Fe–N*_x_* ISAs/GHSs. a) XRD pattern, b) Raman spectra of Fe–N*_x_* ISAs/GHSs and pristine GO nanosheets, c) XPS survey spectrum, d) C 1s region, e) N 1s region, and f) Fe 2p region.

X‐ray diffraction (XRD) pattern (**Figure** **3**a) suggests that the as‐prepared Fe–N*_x_* ISAs/GHS sample only possesses two broad peaks at 25.5° and 42.2°, which are attributable to the (002) and (100) planes of the graphitic carbon, respectively. Importantly, no diffraction peaks related to Fe‐based species emerges, proving that Fe atoms are atomically dispersed in the Fe–N*_x_* ISAs/GHSs. As revealed by the XRD pattern shown in Figure S4 in the Supporting Information, the direct pyrolysis of FePc molecules at 700 °C without any carbon support produces Fe‐based mixture and carbon nitride species. While being loaded on 2D graphene nanosheets, FePc molecules also transform into plenty of Fe‐based nanoparticles after the carbonization treatment, as evidenced by the small diffraction peaks in the XRD pattern (Figure S5a, Supporting Information) and the TEM image (Figure S5b, Supporting Information). Moreover, due to the large surface area and high flexibility, the 2D FePc‐decorated graphene tend to stack and aggregate severely during pyrolysis, providing more collision/sintering possibilities of loaded FePc molecules and thus generating Fe‐based nanoparticles. While in case of 3D graphene hollow nanospheres, the spherical architecture could not only effectively minimize the stack and corrugation of graphene nanosheets, but also inhibit the agglomeration and sintering of the absorbed FePc molecules due to the steric‐hindrance effect caused by the unique spherical architecture. Therefore, 3D graphene hollow nanospheres represent an ideal support to stabilize the single‐atom active sites. Raman spectra of pristine GO nanosheets and Fe–N*_x_* ISAs/GHSs in Figure 3b exhibit two well‐defined peaks, the D band at ≈1366 cm^−1^ ascribed to defects and disordered carbon and G band at ≈1613 cm^−1^ corresponding to sp^2^‐hybridized carbon. Obviously, the Fe–N*_x_* ISAs/GHSs show a lower ratio of the D band to G band intensity (*I*_D_/*I*_G_), indicative a well‐crystallized graphitic carbon and high conductivity of the Fe–N*_x_* ISAs/GHSs.^31^ The N_2_ adsorption–desorption isotherms (Figure S6, Supporting Information) of the Fe–N*_x_* ISAs/GHSs can be classified as type‐IV curves, suggesting the presence of mesopores. Moreover, the rapid N_2_ uptake at *p*/*p*_0_ > 0.9 region indicates the existence larger macropores ascribed to the hollow interior of the graphene nanospheres. The Brunauer–Emmett–Teller surface area of the Fe–N*_x_* ISAs/GHSs is determined to be 375.0 m^2^ g^−1^.

X‐ray photoelectron spectroscopic (XPS) measurements were conducted to acquire further insight into the surface components of the Fe–N*_x_* ISAs/GHSs. The survey spectrum (Figure 3c) suggests the presence of C (95.03 at%), N (3.78 at%), and Fe (1.18 at%). The high‐resolution C 1s spectrum (Figure 3d) shows that Fe–N*_x_* ISAs/GHSs consist of prominent sp^2^‐hybridized carbon (54.07%), along with some nitrogen‐bonded carbon (36.11%) and oxidized carbon (9.82%). The high‐resolution N 1s region (Figure 3e) can be well deconvoluted into four peaks, corresponding to pyridinic N and/or Fe–N (398.4 eV), pyrrolic N (399.8 eV), graphitic N (400.8 eV), and oxidized N (402.4 eV), respectively. It is well documented that pyridinic N could coordinate with Fe atoms to form Fe–N–C structure with optimized local electronic structure and graphitic–N moieties may account for the high activity and stability of the Fe–N–C electrocatalysts. Clearly, both pyridinic N and graphitic N are predominant configurations in the synthesized Fe–N*_x_* ISAs/GHSs, which not only are beneficial for ORR,^35^ but also offer numerous sites for anchoring single Fe atoms. The Fe 2p spectrum (Figure 3f) exhibits two major peaks at 711.4 and 724.8 eV along with minor shakeup satellites, further indicating that Fe might coordinate with N to form Fe–N*_x_* species.^36^

### 2.2 Electrocatalytic Performance for the ORR

Due to the highly steady hollow nanosphere substrate and atomically dispersed Fe sites, the resultant Fe–N*_x_* ISAs/GHSs are expected to be a promising cost‐effective ORR electrocatalyst with high activity and stability. To evaluate the ORR activity of the prepared Fe–N*_x_* ISAs/GHSs, cyclic voltammetry (CV) measurements were initially performed in N_2_ and O_2_‐saturated 0.1 m KOH solutions, respectively. Since the pyrolysis temperature is generally considered as one of the most critical parameters for carbon‐based nanomaterials, the ORR activities of Fe–N*_x_* ISAs/GHSs prepared at different temperatures (600, 700, 800, and 900 °C) were initially studied. As shown in Figure S7 in the Supporting Information, the products obtained at different pyrolysis temperatures share the identical morphological features, i.e., hollow spherical architecture with thin wall. Furthermore, no nanoparticles can be observed from the TEM images of different samples, indicating the atomic dispersion of Fe sites. From the XRD pattern (Figure S8a, Supporting Information), all samples obtained at different temperatures exhibit two broad peaks corresponding to the (002) and (100) planes of the graphitic carbon. Additionally, no diffraction peaks ascribed to Fe‐based species can be observed for all samples. As shown in Figure S9 in the Supporting Information, the product prepared at 700 °C exhibits the most positive onset potential (*E*_onset_) and half‐wave potential (*E*_1/2_) among all catalysts, indicating the best ORR activity. This observation is possibly resulted from the fact that lower pyrolysis temperature (600 °C) may produce a poor graphitization degree and thus an unsatisfactory conductivity, whereas too much higher pyrolysis temperature (800 and 900 °C) would destroy the graphic structure and lead to the formation of defective structure, as evidenced by the Raman spectra (Figure S8b, Supporting Information). Therefore, 700 °C represents an optimal pyrolysis temperature in the present synthetic protocol and the Fe–N*_x_* ISAs/GHSs discussed below are prepared at the pyrolysis temperature of 700 °C. For comparison, commercial 20 wt% Pt/C catalyst and 2D FePc/GO nanosheets‐derived product (denoted as Fe–N*_x_*/GNSs, Figure S5, Supporting Information) were used as reference samples tested under the identical conditions.

**Figure 4:**
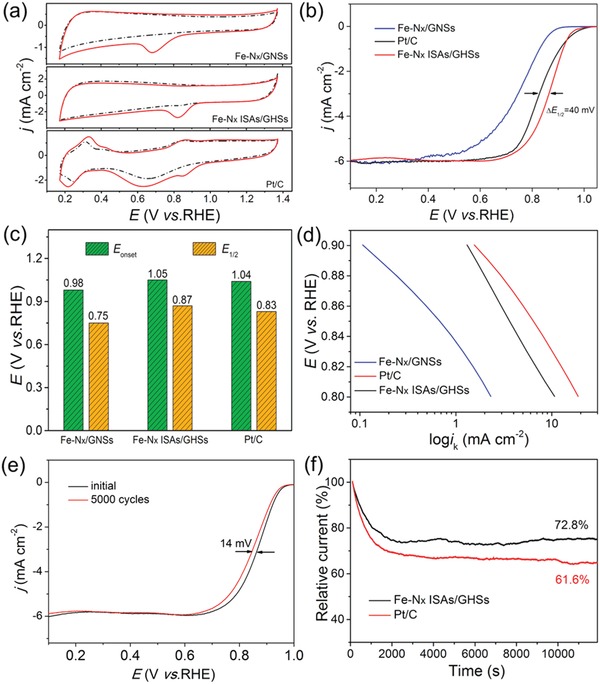
Comparison of ORR performances of Fe–N*_x_*/GNSs, Fe–N*_x_* ISAs/GHSs, and commercial Pt/C. a) CV curves recorded in O_2_‐saturated (solid line) or N_2_‐saturated (dashed line) in 0.1 m KOH solution, b) LSV polarization curves recorded in O_2_‐saturated in 0.1 m KOH with a rotation rate of 1600 rpm, c) *E*_onset_ and *E*_1/2_ of different catalysts, d) Tafel plots, e) ORR polarization curves of the Fe–N*_x_* ISAs/GHSs before and after 5000 cycles, f) *i*–*t* curves of Fe–N*_x_* ISAs/GHSs and Pt/C in O_2_‐saturated in 0.1 m KOH solution at 0.6 V.

As shown in **Figure** **4**a, in comparison with the featureless CV curves recorded in N_2_‐saturated electrolyte, well‐defined cathodic peaks appear at around 0.69, 0.83, and 0.85 V for Fe–N*_x_*/GNSs, Fe–N*_x_* ISAs/GHSs and commercial Pt/C, respectively, in O_2_‐saturated solution, signifying a pronounced ORR activity of Fe–N*_x_* ISAs/GHSs. To establish the outstanding ORR activity of the Fe–N*_x_* ISAs/GHSs, we further performed rotating disk electrode (RDE) measurements in O_2_‐saturated 0.1 m KOH solution with a scan rate of 5 mV s^−1^ and a rotating rate of 1600 rpm. The linear sweep voltammetry (LSV) curves (Figure 4b) clearly demonstrate that Fe–N*_x_* ISAs/GHSs exhibit the highest activity with an impressive *E*_onset_ and the most positive *E*_1/2_ among the three catalysts. Specifically, as illustrated in Figure 4c, the *E*_onset_ on Fe–N*_x_* ISAs/GHSs is observed to be 1.05 V, which is very close to that of commercial Pt/C (1.04 V) and much higher than that of Fe–N*_x_*/GNSs (0.98 V). Additionally, the Fe–N*_x_* ISAs/GHS catalyst exhibits an *E*_1/2_ of 0.87 V, which is more positive than those of commercial Pt/C (0.83 V) and Fe–N*_x_*/GNSs (0.75 V). The above results unambiguously indicate that the resultant Fe–N*_x_* ISAs/GHSs possess a superior ORR activity to commercial Pt/C in alkaline medium. Notably, our Fe–N*_x_* ISAs/GHSs could exhibit competitive or even better ORR activity in alkaline medium with relatively positive *E*_onset_ and *E*_1/2_ in comparison with the previously reported Fe–N–C ‐based ORR electrocatalysts, as briefly listed in Table S1 in the Supporting Information. The Tafel curves (Figure 4d) derived from the LSV curves indicate that Fe–N*_x_* ISAs/GHSs could afford the largest kinetic current density compared with the other two reference samples in a potential range of 0.80–0.90 V, further confirming the superior activity of Fe–N*_x_* ISAs/GHSs. Since the loading amount of Fe is quite low in the synthesized Fe–N*_x_* ISAs/GHSs, the greatly enhanced ORR activity strongly manifests the high efficiency of atomically dispersed active sites. To further identify the ORR pathway of the prepared Fe–N*_x_* ISAs/GHSs, rotating ring‐disk electrode (RRDE) measurement was performed to monitor the H_2_O_2_ yield, as shown in Figure S10 in the Supporting Information. Evidently, the disk current density is remarkably larger than the ring current density and the H_2_O_2_ yield is relatively low (<5%), indicative the formation of predominant OH^−^ species during the ORR. The electron transfer number (*n*) is determined to be approximately 4, implying a desirable 4‐electron pathway over the single Fe sites. Accelerated durability tests suggest that the Fe–N*_x_* ISAs/GHSs exhibit an excellent durability. As shown in Figure 4e, Fe–N*_x_* ISAs/GHSs show a minor deterioration with only 14 mV negative shift of *E*_1/2_ after 5000 continuous potential cycles, which is related to the fact that the atomically dispersed Fe atoms are firmed anchored by the unique phthalocyanine‐like structures on the highly stable graphene hollow spheres. Additionally, TEM images (Figure S11, Supporting Information) indicate that the hollow and spherical feature of the Fe–N*_x_* ISAs/GHSs can be perfectly maintained after the stability test. Furthermore, AC‐HAADF‐STEM image demonstrates that Fe atoms were still atomically dispersed on the hollow graphene nanospheres, suggesting their superb structural robustness. In contrast, the *E*_1/2_ of commercial Pt/C negatively shifts approximately 42 mV under the identical condition (Figure S12, Supporting Information), which may be caused by the agglomeration of Pt nanoparticles after the ORR, as revealed by the TEM images (Figure S13, Supporting Information).

The stability of the Fe–N*_x_* ISAs/GHSs was further confirmed by chronoamperometry (CA) measurements at 0.6 V in O_2_‐saturated solution, as shown in Figure 4f. After 12 000 s of continuous operation, the Fe–N*_x_* ISAs/GHSs exhibit 72.8% retention of the initial current density, which is significantly higher than that observed on commercial Pt/C (61.6%).

The outstanding ORR performance of the resultant Fe–N*_x_* ISAs/GHSs can be reasonably attributed to their structural advantages. Specifically, 1) the atomically dispersed Fe–N*_x_* species could effectively maximize the atomic utilization efficiency and also guarantee the homogeneity of active sites.^32^, ^37^, ^38^ 2) The hollow graphene nanospheres with high mechanical strength not only provide a rigid and robust scaffold for anchoring single‐atom active sites, but also prevent the stacking and corrugation of graphene nanosheets, facilitating to increase the structural stability.^25^, ^39^ 3) The hollow nanospheres with open mouths, hollow cavities, and exposed interior surface could be beneficial for mass transfer and diffusion, expediting the reaction kinetics.^40^, ^41^ All aforementioned favorable features render the Fe–N*_x_* ISAs/GHSs an efficient ORR electrocatalyst with enhanced activity and stability.

**Figure 5:**
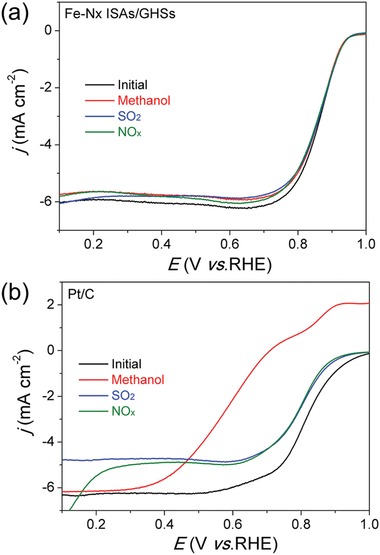
Antipoisoning tests of a) Fe–N*_x_* ISAs/GHSs and b) commercial Pt/C for ORR in the presence of methanol, SO_2_, and NO*_x_*.

As well known, antipoisoning capability of an electrocatalyst is also of great importance for practical applications. It is well documented that, in direct methanol fuel cells, the cathodic ORR of Pt‐based nanocatalysts usually suffer from the crossover effect caused by methanol from the anode, resulting in a declining cell performance. In addition, even trace SO_2_ and NO*_x_* in air may also poison the Pt‐based nanocatalysts, significantly reducing the activity and stability of air‐breathing fuel cells. In order to investigate their antipoisoning capabilities, ORR polarization curves of the fabricated Fe–N*_x_* ISAs/GHSs and commercial Pt/C were recorded in O_2_‐saturated KOH solution in the presence of methanol (0.5 m), SO_2_ (in the form of 0.01 m Na_2_SO_3_), and NO*_x_* (in the form of 0.1 m NaNO_2_). As shown in **Figure** **5**a, the LSV curves of Fe–N*_x_* ISAs/GHSs remain almost unchanged even upon the introduction of methanol, SO_2_, and NO*_x_*, demonstrating the high selectivity toward the ORR with strong tolerance to poisonous species. Such high tolerance capability of our Fe–N*_x_* ISAs/GHSs may origin from the preferential adsorption of O_2_ over methanol, SO_2_, and NO*_x_* on the Fe sites of the resultant Fe–N*_x_* ISAs/GHSs.^37^ In a striking contrast, commercial Pt/C catalyst shows a remarkable attenuation in ORR activity with largely negative shift of *E*_1/2_ under the identical conditions (Figure 5b). The above results manifest that the Fe–N*_x_* ISAs/GHSs may hold great promises in fuel cell devices with lower cost, higher activity, and better selectivity.

## 3 Conclusion

In summary, we have demonstrated a rational design and synthesis of atomically dispersed Fe atoms anchored on highly stable hollow graphene nanospheres through a template‐engaged approach by using FePc as Fe source. The rigid planar macrocycle structure of FePc molecules and π‐π interaction between FePc and graphene nanosphere substrate account for the formation of isolated Fe single atomic sites. Benefiting from the homogeneously dispersed atomic active sites, high porosity, and excellent robustness of graphene nanosphere substrate, the resultant Fe–N*_x_* ISAs/GHSs exhibit extraordinary ORR activity, long‐term stability, and high tolerance to methanol, SO_2_, and NO*_x_*, outperforming commercial Pt/C catalyst and most of previously reported Fe‐based catalysts in alkaline medium. The enhanced ORR performance makes the synthesized Fe–N*_x_* ISAs/GHSs high‐efficiency and cost‐effective electrocatalysts for fuel cells and metal–air batteries. The present work not only affords a promising non‐precious ORR electrocatalyst to substitute Pt, but also provides a general and versatile strategy to prepare other metal single atoms stabilized on carbon substrates for diverse electrocatalytic applications.

## 4 Experimental Section

*Reagents and Chemicals*: Poly(diallyldimethylammonium chloride) (*M*_w_ < 500 000 Da) and poly(sodium4‐styrenesulfonate) (*M*_w_ < 700 000 Da) were purchased from Alfa Aesar Co. Ltd. Graphene oxide (GO) was supplied by Nanjing XFNANO Materials Tech Co. Ltd. (Nanjing, China). Tetraethylorthosilicate and NaOH were purchased from Sinopharm Chemical Reagent Co. Ltd. (Shanghai, China). Commercial 20 wt% Pt/C was purchased from Johnson Matthey Corporation. Dimethyl formamide (DMF) was purchased from Sigma–Aldrich and used as received. All the reagents were of analytical reagent grade and used without further purification.

*Synthesis of Fe–N_x_ ISAs/GHSs*: For the synthesis of Fe–N*_x_* ISAs/GHSs, uniform SiO_2_ nanospheres (160 nm in diameters) were first prepared via a modified stöber method and served as sacrificial templates to define the morphology and size of the Fe–N*_x_* ISAs.^42^ Subsequently, the preformed SiO_2_ nanospheres were encapsulated with GO nanosheets according to our previous protocol, forming SiO_2_@GO nanospheres (*W*_SiO2_/*W*_GO_ = 4:1). Afterward, 100 mg of the prepared SiO_2_@GO nanospheres were dispersed into 20 mL of deionized water with continuously stirring. Meanwhile, 5 mg of FePc,^43^ which was synthesized according to the literatures, was dispersed in 20 mL of DMF solution. Then the FePc/DMF solution was added into the above SiO_2_@GO nanosphere suspension and kept stirring for 5 h, forming SiO_2_@GO/FePc nanospheres. The resultant SiO_2_@GO/FePc nanospheres were annealed at 700 °C for 3 h under a N_2_ atmosphere, transforming FePc molecules into atomically dispersed Fe–N*_x_* species. Meanwhile, GO deoxidized to reduced GO under 700 °C. Finally, the SiO_2_ nanosphere templates were etched away by 20 mL of 2 m NaOH solution for 24 h, leading to the formation of Fe–N*_x_* ISAs/GHSs.

For comparison, 2D graphene nanosheets supported Fe–N*_x_* sample (denoted as Fe–N*_x_*/GNSs) were synthesized applying the similar approach above by using 20 mg of 2D GO nanosheets as supports, instead of 100 mg of SiO_2_@GO nanospheres.

*Characterization*: XRD analyses were conducted on a Model D/max‐rC X‐ray diffractometer employing Kα radiation (λ = 0.15406 nm). TEM images and high‐resolution TEM (HRTEM) images were acquired from JEOL JEM‐2010 instrument at an accelerating voltage of 200 kV. The AC‐HAADF‐STEM images were performed on JEOL JEM‐ARM 200F. SEM images were taken from a Hitachi S‐4800 microscopy. Raman spectra were recorded on a Raman spectrometer (LabRAM HR800, λ = 514 nm). XPS measurements were carried out on a Thermo VG Scientific ESCALAB 250 spectrometer with an Al Kα radiator. The binding energy was calibrated by means of the C 1s peak energy of 284.6 eV. N_2_ adsorption/desorption isotherms were measured on thermo fisher scientific surfer gas adsorption porosimeter.

*Electrochemical Measurements*: All electrochemical tests were carried out on a CHI 760D electrochemical analyzer (Shanghai, Chenghua Co.) equipped with high‐speed rotators from gamry instruments. A conventional three‐electrode system was used, including an RDE or RRDE as the working electrode (5 mm in diameter, 0.196 cm^2^), a Pt wire as the auxiliary electrode, and a saturated calomel electrode (SCE) as the reference electrode. For electrode preparation, 5 mg of as‐prepared electrocatalyst was sonicated in a solution containing 0.9 mL ethanol and 0.1 mL neutralized Nafion (5 wt%, Sigma–Aldrich) for 30 min to form a homogeneous catalyst ink. A 10 µL aliquot of the catalyst ink was loaded on the polished RDE or RRDE and dried at room temperature. CV tests were carried out in N_2_‐saturated 0.1 m KOH solution. Before ORR test, pure oxygen gas (99.9%) was purged for 30 min to make the electrolyte saturated with O_2_. The RDE and RRED measurements were conducted in O_2_‐saturated 0.1 m KOH electrolyte at a rotation rate of 1600 rpm with a scan rate of 5 mV s^−1^. All potentials in this paper were converted to potentials versus the reversible hydrogen electrode (RHE), using the following equation: *E*_RHE_ = *E*_SCE_ + 0.0591 pH + 0.242. The percentage of HO_2_^−^ intermediate production (%HO_2_^−^) and electron transfer number (*n*) were determined according to the followed equations:^44^

$$\%{\mathrm{HO}}_{2}^{-}=\frac{200I_{r}}{NI_{d}+I_{r}}\;n=\frac{4NI_{d}}{NI_{d}+I_{r}}$$

where *I*_d_ is the disk current, *I*_r_ is the ring current, and *N* is the current collection efficiency of the Pt ring, which was determined to be 0.37.

## Conflict of Interest

The authors declare no conflict of interest.

## Supporting information

Supplementary
