# Supplementary material for: Isolated Fe Single Atomic Sites Anchored on Highly Steady Hollow Graphene Nanospheres as an Efficient Electrocatalyst for the Oxygen Reduction Reaction
Source: Adv Sci (Weinh). 2018 Nov 26;6(2):1801103. doi: 10.1002/advs.201801103 (PMC6343057; doi:10.1002/advs.201801103)
Supplement: Supplementary file 1 — Supplementary [file ADVS-6-1801103-s001.pdf]

Copyright WILEY-VCH Verlag GmbH & Co. KGaA, 69469 Weinheim, Germany, 2018.

ADVANCED SCIENCE
Open Access

Supporting Information

for *Adv. Sci.,* DOI: 10.1002/advs.201801103

Isolated Fe Single Atomic Sites Anchored on Highly Steady Hollow Graphene Nanospheres as an Efficient Electrocatalyst for the Oxygen Reduction Reaction

Xiaoyu Qiu, Xiaohong Yan, Huan Pang, Jingchun Wang, Dongmei Sun, Shaohua Wei, Lin Xu, and Yawen Tang**

Supporting Information

Isolated Fe Single Atomic Sites Anchored on Highly-steady Hollow Graphene Nanospheres as an Efficient Electrocatalyst for the Oxygen Reduction Reaction

Xiaoyu *Qiu‡, Xiaohong Yan‡, Huan Pang*, Jingchun Wang, Dongmei Sun, Shaohua Wei, Lin Xu, * and Yawen Tang *

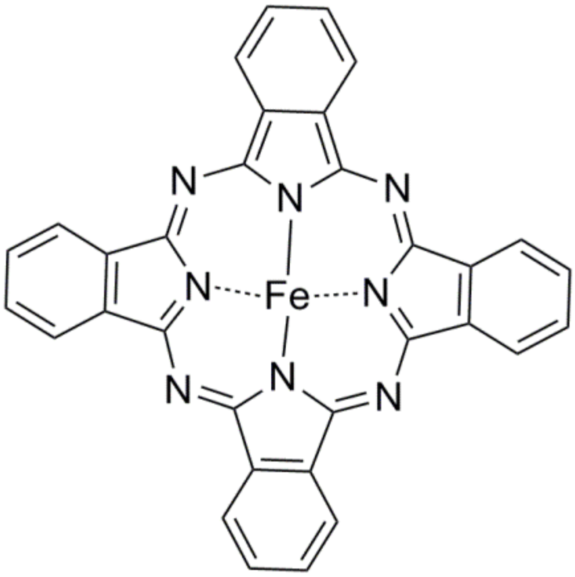

Figure S1 Molecular structure of iron phthalocyanine (FePc).

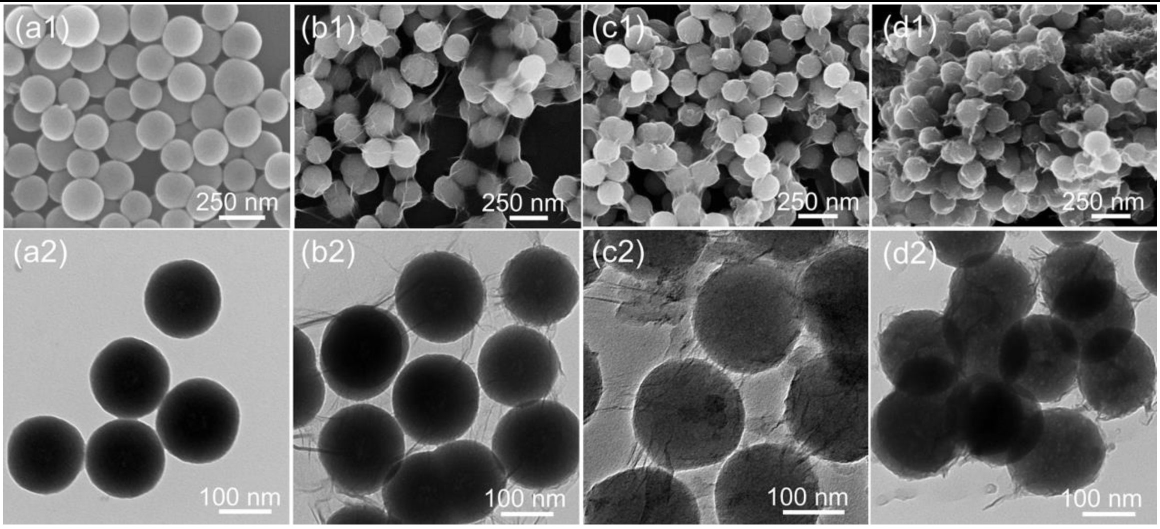

Figure S2 TEM and SEM images of the intermediate products. (a1)-(a2) SiO_2 nanospheres, (b1)-(b2) SiO_2@GO nanospheres, (c1)-(c2) SiO_2@GO/FePc nanospheres, and (d1)-(d2) SiO_2@rGO/ Fe-N_x ISAs nanospheres.

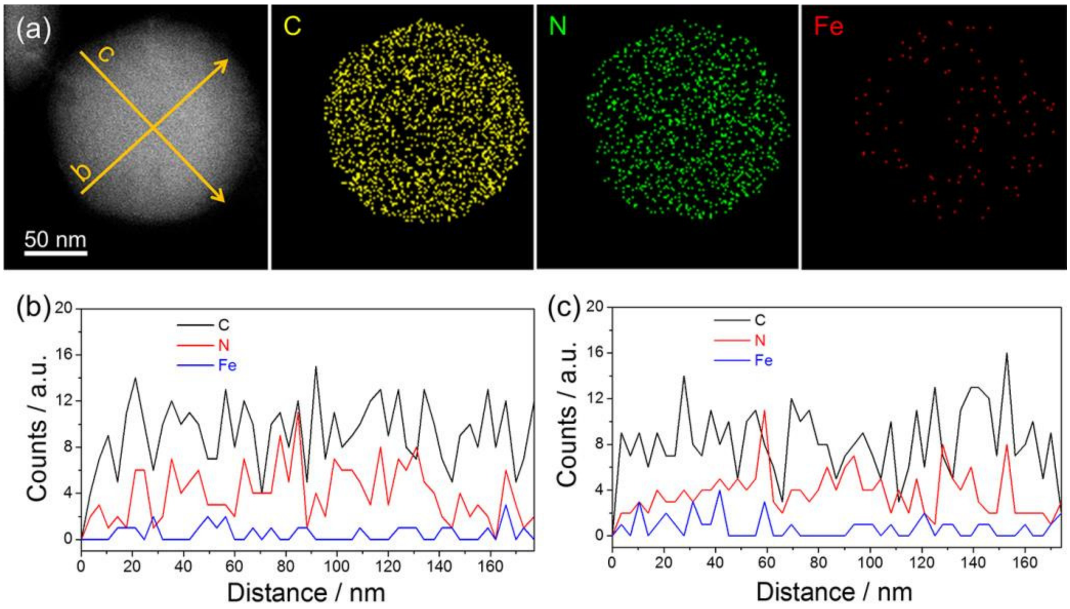

Figure S3 (a) HAADF-STEM image and elemental mapping images, and (b)-(c) EDX line-scan profiles of the SiO_2@GO/FePc nanospheres.

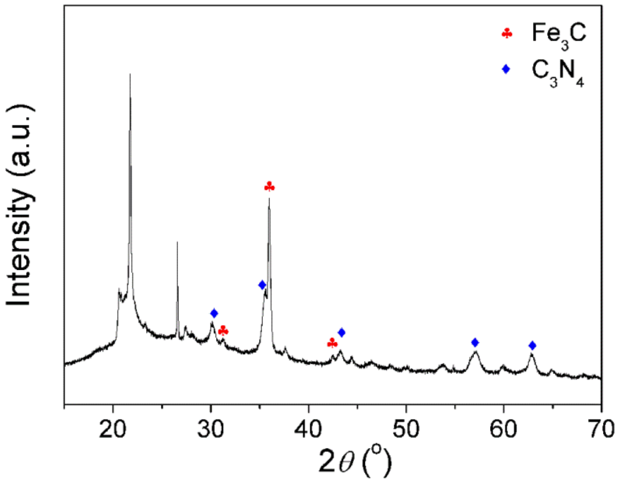

Figure S4 XRD pattern of the product derived from the direct pyrolysis of FePc molecules.

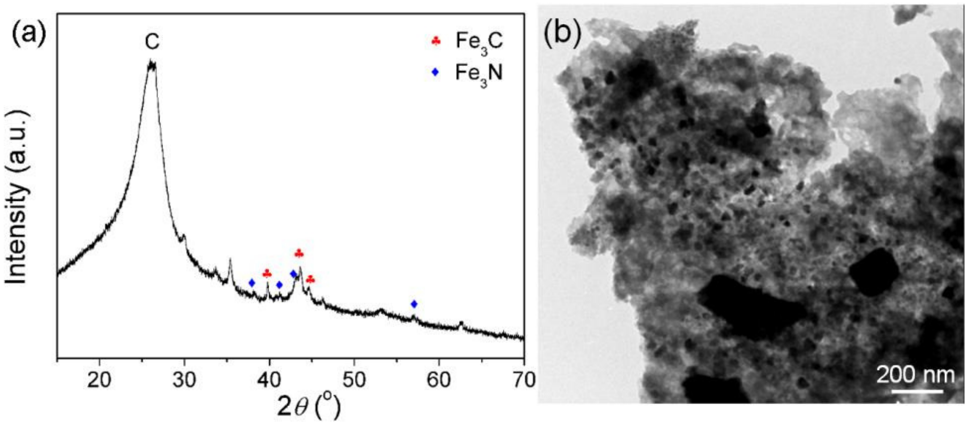

Figure S5 (a) XRD pattern and (b) TEM image of the FePc/GO nanosheets-derived product (denoted as Fe-N_x/GNSs).

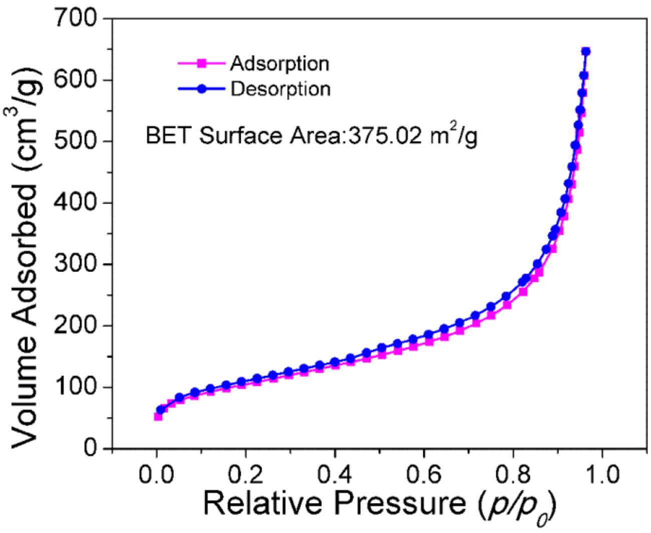

Figure S6 N_2 adsorption-desorption isotherms of Fe-N_x ISAs/GHSs.

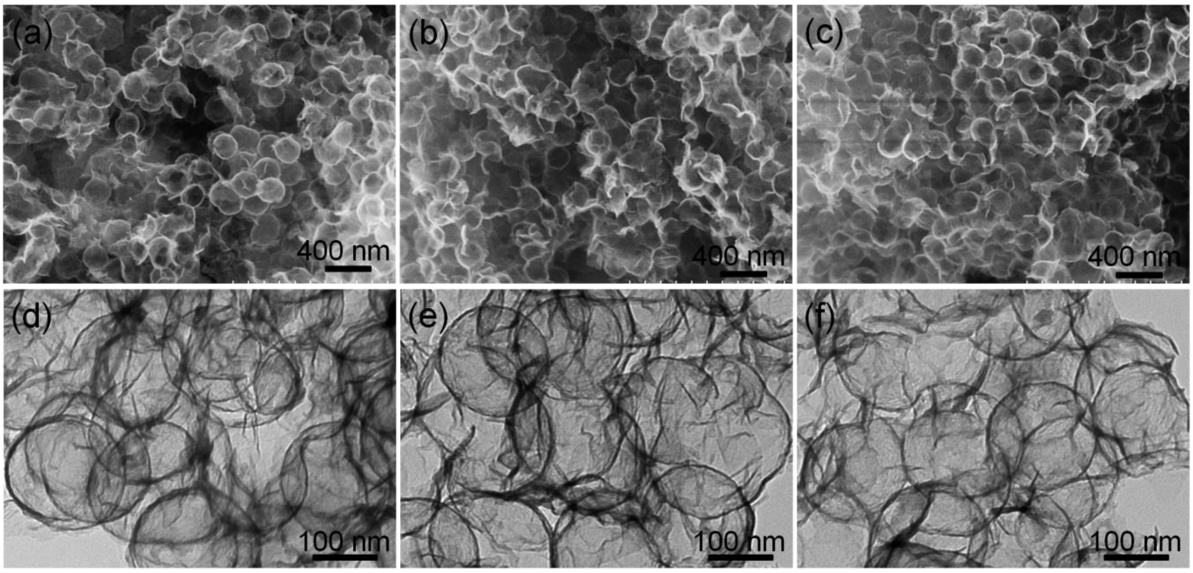

Figure S7 TEM and SEM images of the products prepared at different temperatures. (a)-(d) 600 ^{o}C, (b)-(e) 800 ^{o}C and (c)-(f) 900 ^{o}C.

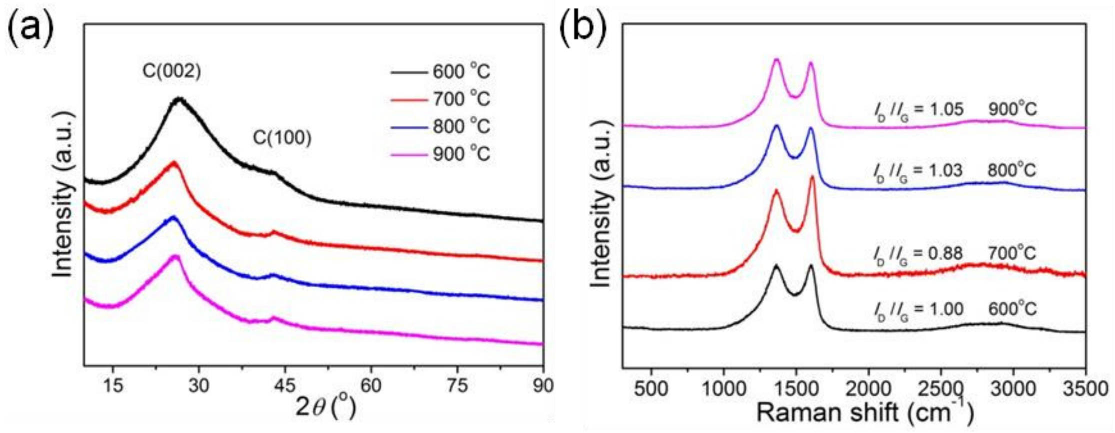

Figure S8 (a) XRD patterns and (b) Raman spectra of the products prepared at different pyrolysis temperature.

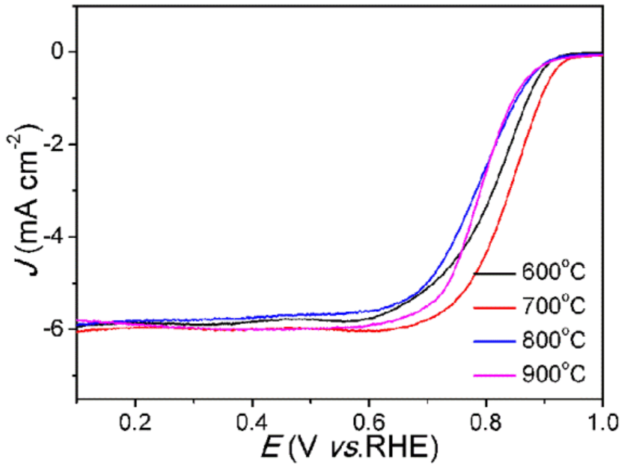

Figure S9 ORR polarization curves of the Fe-N_x ISAs/GHSs prepared at different pyrolysis temperatures in O_2-saturated 0.1 M KOH solution at a sweep rate of 5 mV s^{-1} at 1600 rpm.

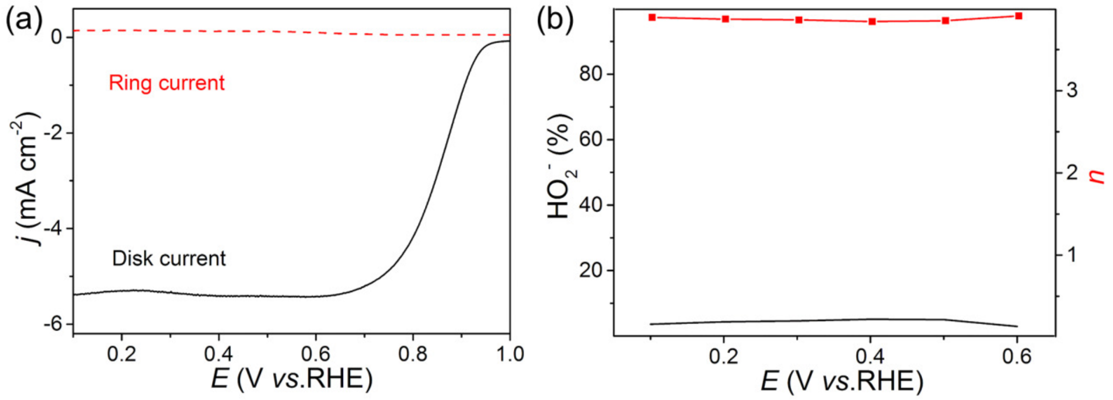

Figure S10 (a) RRDE test of the Fe-N_x ISAs/GHSs in O_2-saturated 0.1 M KOH solution at a sweep rate of 5 mV s^{-1} at 1600 rpm. (b) HO_2^- yield and electrons transfer number n of the Fe-N_x ISAs/GHSs during the RRDE test.

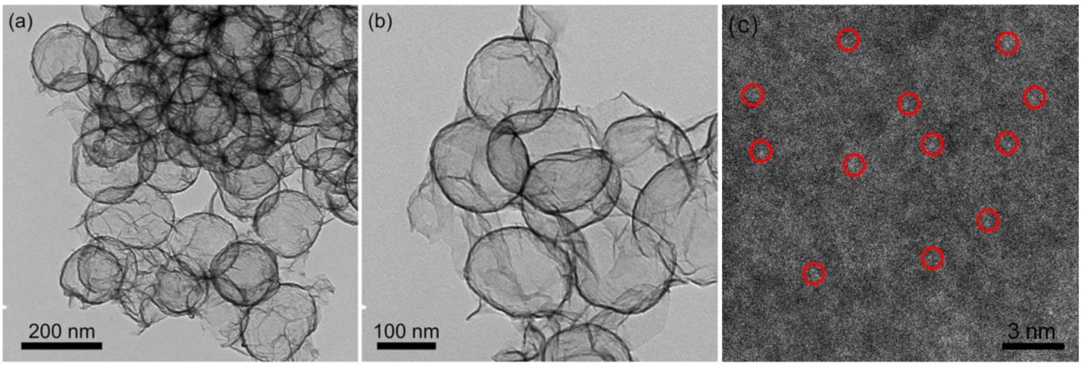

Figure S11 (a)-(b) TEM images and (c) aberration corrected HAADF-STEM image of the Fe-N_x ISAs/GHSs after the stability test.

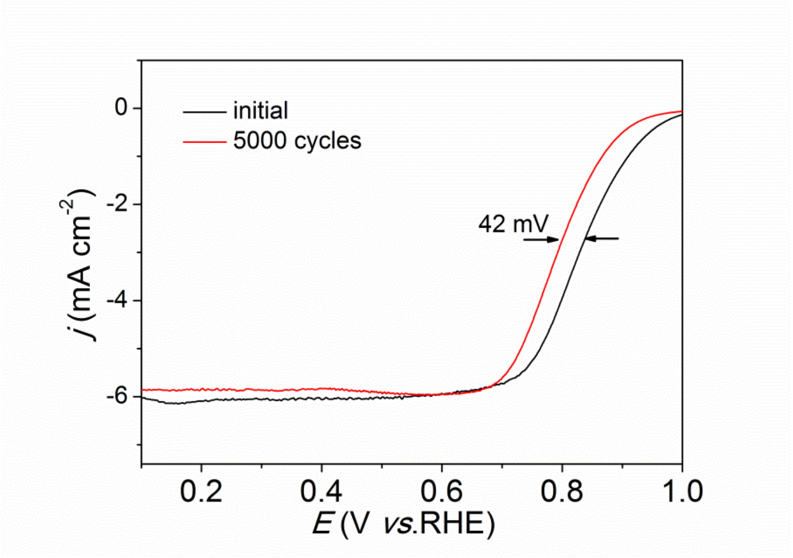

Figure S12 ORR polarization curves of the Pt/C before and after 5000 cycles.

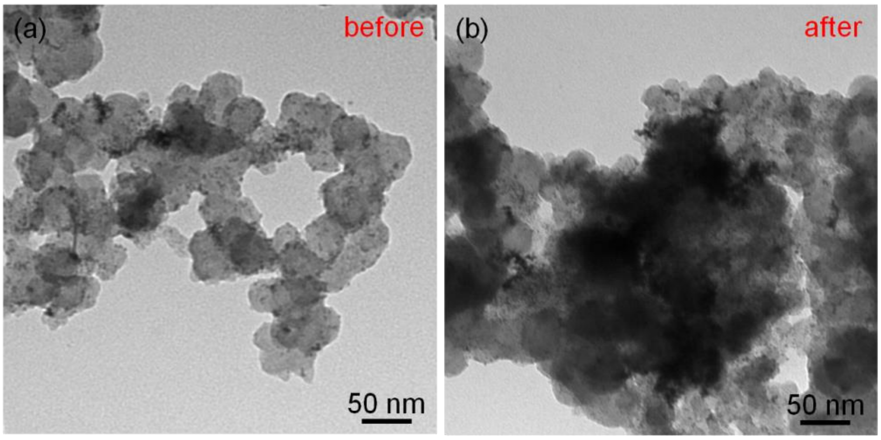

Figure S13 TEM images of the Pt/C (a) before and (b) after the stability test.

Table S1. Comparison of the ORR performance of the synthesized Fe-N_x ISAs/GHSs with some previously reported non-precious catalysts in KOH solution.

Number	Electrocatalysts	E_{onset} / V vs. RHE	$E_{1/2}$ / V vs. RHE	Ref
	Fe-N_x ISAs/GHSs	1.05	0.87	This work
1	p-Fe-N-CNFs	0.94	0.82	Energy Environ. Sci. 2018, DOI: 10.1039/c8ee00673c
2	FeSAs/PTF-600	1.01	0.87	ACS Energy Lett. 2018, 3, 883.
3	Atomic Fe−NGM/C−Fe	1.05	0.86	Chem. Mater. 2017, 29, 9915.
4	Fe−N-doped DSC	1.025	0.833	ACS Nano 2018, 12, 208.
5	pCNT@Fe@GL/CNF	N. A.	0.811	Adv. Mater. 2017, 1606534.
6	Fe–N-CNTAs-5-900	0.970	0.880	Small 2017, 13, 1603407.
7	Fe-N/MC@0.1	0.990	0.850	ChemCatChem 2015, 7, 2937.
8	Fe-ISAs/CN	0.990	0.900	Angew. Chem. Int. Ed. 2017, 129, 7041.
9	Graphene-like macroporous Fe-N-C Catalyst	N. A.	0.88	ACS Catal. 2017, 7, 6144.
10	Fe,N-doped carbon nanofibers	0.98	0.83	Chem. Mater. 2017, 29, 5617.
11	Fe/N-doped carbon nanofibers	0.88	0.79	Small 2016, 12, 6398.
12	Fe-N-doped mesoporous carbon microspheres	1.027	0.86	Adv. Mater. 2016, 28, 7948.
13	FeCo, N-codoped Porous Carbon Networks	1.050	0.88	Small 2016, 12, 4193.
14	Fe_2N/mesoporous N-doped graphitic carbon spheres	0.95	0.87	Nano Energy 2016, 24, 121.

15	Fe_3C nanoparticle embedded mesoporous carbon	1.02	0.86	Small 2016, 12, 5414.
16	C-FeZIF-900-0.84	0.950	0.860	ACS Appl. Mater. Interface. 2017, 9, 9699.
17	S,N-Fe/N/C-CNT	0.960	0.850	Angew. Chem. Int. Ed. 2017, 56, 610.
18	Fe-N/C-700 nanosheets	0.956	0.84	Small 2016, 12, 5710.
